# A word-count approach to analyze linguistic patterns in the reflective writings of medical students

**DOI:** 10.3402/meo.v21.29522

**Published:** 2016-02-01

**Authors:** Chi-Wei Lin, Meei-Ju Lin, Chin-Chen Wen, Shao-Yin Chu

**Affiliations:** 1 Department of Counseling & Clinical Psychology, Dong Hwa University, Taiwan; 2 Department of Human Development, Tzu Chi University, Taiwan; 3 Department of Pediatrics, Buddhist Tzu Chi General Hospital, Taiwan; 4 School of Medicine, Tzu Chi University, Taiwan

**Keywords:** linguistic pattern, reflective writing, medical education, health professions education

## Abstract

**Background:**

Teaching reflection and administering reflective writing assignments to students are widely practiced and discussed in medical education and health professional education. However, little is known about how medical students use language to construct their narratives. Exploring students’ linguistic patterns in their reflective writings can facilitate understanding the scope and facets of their reflections and their representational or communication approaches to share their experiences. Moreover, research findings regarding gender differences in language use are inconsistent. Therefore, we attempted to examine how females and males differ in their use of words in reflective writing within our research circumstance to detect the unique and gender-specific approaches to learning and their applications.

**Methods:**

We analyzed the linguistic profiles of psychological process categories in the reflective writings of medical students and examined the difference in word usage between male and female medical students. During the first year of a clinical rotation, 60 fifth-year medical students wrote reflective narratives regarding pediatric patients and the psychosocial challenges faced by the patients and their family members. The narratives were analyzed using the Chinese version of Linguistic Inquiry and Word Count (CLIWC), a text analysis software program. Multivariate procedures were applied for statistical analysis.

**Results:**

Cognitive words were most pervasive, averaging 22.16%, whereas perceptual words (2.86%) were least pervasive. Female students used more words related to positive emotions and sadness than did male students. The male students exceeded the female students only in the space category. The major limitation of this study is that CLIWC cannot directly acquire contextual text meanings; therefore, depending on the research topic, further qualitative study of the given texts might be necessary.

**Conclusions:**

To enhance students’ empathy toward the psychosocial issues faced by patients and their family members, students should be encouraged to explore the domain of psychological processes by identifying and expressing their affective and perceptual experiences. Researchers in future studies should use outcome measures such as self-awareness or empathy to determine the overall effectiveness of reflective writing and how changes in linguistic patterns affect such outcomes.

Reflective writing, defined as an expertise-enhancing metacognition process, has been demonstrated to be a valuable educational tool for helping learners develop their critical thinking skills, challenge facile assumptions, facilitate empathy, and prevent disillusionment (1, 2). Such writing can affect transformative outcomes from reflecting on experience and can be used to validate existing experience (3, 4).

The importance of reflection and reflective practice for medical education and across various health professions is frequently noted in the literature (5). Among various reflective techniques, several forms of reflective writing aimed at enhancing self-awareness and gaining an understanding of the perspectives of others have been created, and such forms include learning journals (6) and memos (7). However, little is known about how students use language to construct narratives. As claimed by Tausczik and Pennebaker (8), language is the most common and reliable method to translate internal thoughts and emotions to others. The words people use reflect not only their attention and attitude in daily life but also how they perceive the world. Moreover, Charon and Hermann (9) reported that reflection can be considered an active inner state that entails using cognitive and affective means to perceive one's experience and represent it in language. Determining how students use language to construct their narratives can facilitate gaining a clearer understanding of the scope and facets of their reflections and their representational or communication approaches. Studying the word constructions of students’ reflective writings enables comprehensively measuring of the following aspects: the scope of the students’ focus, the students’ cognitive processing and individual particularities that facilitate their self-awareness, and how students structure their learning experience with patients. These aspects provide a valuable basis for processes sensitizing students’ continuous reflection on their learning and clinical experience.

Linguistic Inquiry and Word Count (LIWC), a computerized text analysis software program, is an appropriate tool for studying the word constructions of a narrative. Developed originally for measuring cognitive change with health outcomes, this program calculates the percentages of words and terms in a text that fall into certain categories (10). Accordingly, LIWC provides comprehensive quantitative measures that operationalize aspects of cognitive processing and perceptual orientations.

In the current study, overall language patterns and gender differences were explored using the Chinese version of Linguistic Inquiry and Word Count (CLIWC). According to Tausczik and Pennebaker (8), natural language use reveals people's disposition regarding their processing and interpretation of information to make sense of the environment. Therefore, differential frequencies among various word categories indicate habitual styles of observing, retrieving, and decoding information. The texts to be analyzed in our study were students’ reflections of the psychosocial matters concerning patients and their families. On the basis of the theme and scope of this study, six psychological process word categories (social, affective, cognitive, perceptual, biological, and relativity) and their subcategories were coded and analyzed (explained in detail in the Instrument section). By using CLIWC in analyzing students’ reflective writings, we intended to determine how students attend to the presentation of patients and how they represent and process their interaction with them. For example, the use of more cognitive words in students’ writings might suggest a stronger tendency to comprehend and analyze their experience, which is beneficial in helping people handle stressful situations, whereas more perceptual words might indicate greater awareness of sensory information, detailing what was seen, heard, and felt.

According to Pennebaker and Graybeal (10), language is a marker of cognitive processes. Studying gender differences in language might therefore reveal unique and gender-specific approaches to learning. In addition, the varying performance, habits, sensitivity, and even the capacity for various self-reflection intensities between genders warrant further study. Previous LIWC studies have reported gender differences in word usage, suggesting that females use social and emotive words more often than males do, whereas males use more complex language than females do (8). However, after an extensive review, Pennebaker et al. (11) concluded that ‘Despite the comparatively large number of studies available on gender differences in language use, no clear picture has yet evolved’ (pp. 557). For example, evidence of gender difference in the use of first-person singular (e.g., ‘I’ and ‘me’) or filler words (e.g., ‘you know’ and ‘like’) is contradictory. Because no clear assessment of gender differences has ensued and because language use depends on situational contexts (8, 11), further contextual and participant-specific studies may provide a clearer picture of whether any difference exists in this respect. On the basis of the literature and the objectives of this study, we used psychological process categories as the objects of comparison. By using CLIWC in the analysis of gender differences in language use, we could empirically examine how female and male students perceived, reflected, and described their experience with patients. Two research questions were addressed: 1) What are the proportions of psychological process words used by students in their reflective writing?; and 2) Are there any differences in the use of these words between the male and female students?

## Methods

### Course and writing assignment as a reflective practice model

Between 2011 and 2012, a compulsory writing course was designed for fifth-year medical students on rotation at the pediatric department of a medical center in eastern Taiwan. This course was designed to help medical students determine the psychosocial issues of patients and their family members. The learning objectives included developing clinical observation skills, empathetic listening skills, interpersonal and communication skills, and problem-solving abilities. The required writing assignment was structured in a narrative, reflective format according to guidelines developed from Gibbs’ concept of a reflective practice model (12). The writing assignment instructions provided to the medical students were the learning objectives of this reflective writing course in addition to orientations on how to use Gibbs's reflective writing model to complete their writing assignments. All students received the same instruction without gender-specific tasks.

### Participants

An entire class of fifth-year medical students participated in this required clinical course. They had the experience of writing a self-reflective diary in the first year of medical school and received writing feedbacks from their mentor. They also had the experience of writing their silent mentor's life story (in group) after interviewing the silent mentor's family members during the 3-year anatomy course.

These medical students completed their writing assignments according to their own schedules and only after meeting and taking medical history from their newly admitted patients. To facilitate diverse thinking, the completed narratives were shared through e-mail before a small group discussion was convened. We coded and analyzed the original texts. Neither e-mail reading nor the group discussion could have confounded the analysis result. However, groups of students may have met by themselves (in the absence of faculty facilitators) to discuss their narratives before they were submitted for the formal group discussion, and those discussions may have influenced their narratives and potentially confounded the findings.

Seventy-two fifth-year students who were enrolled in the present course observed and interacted with patients and their families, reflecting on their experiences by writing narrative accounts. These participants remained anonymous and their personal demographic data were protected. To avoid any possible interference, this study did not influence the students’ grades. After texts, which did not address the situation of patients, were excluded, 60 narrative texts remained for analysis (male: 34; female: 26; mean age=24.7 years).

Students were instructed to follow the courses objectives with a focus on writing patients’ psychosocial issues. The excluded texts may reveal that the writers have not been able to put their concepts into practice or that they were habitually insensitive to psychosocial issues. Reviewing their difficulties in reflecting psychosocial issues in the future is valuable.

### Instrument

In this study, linguistic patterns were coded using CLIWC (13, 14). LIWC involves a word count approach, calculating word frequencies and converting them into percentages for 80 language categories, such as pronouns, and psychological and personal concern categories. Its average classification rate of any given words in various writing and speech texts is 86% (15). Using LIWC is new to the medical field, but it has been used in an increasing number of studies such as student performance evaluations (16), suicide notes (17), and the prediction of neuroticism and depression (18).

According to the design of this study, six psychological process categories and their subcategories were coded and computed. These categories comprised social (e.g., they; subcategories: family, friend, and humans), affective (e.g., happy; subcategories: positive and negative emotions, which comprise anxiety, anger, and sadness), cognitive (e.g., understand; subcategories: insight, causation, discrepancy, tentative, certainty, inhibition, inclusive, and exclusive), perceptual (e.g., heard; subcategories: see, hear, and feel), biological (e.g., blood; subcategories: body, health, sexual, and ingestion), and relativity (e.g., before; subcategories: motion, space, and time). The social process category denoted human interaction (e.g., talking, sharing), whereas the perceptual process represented multiple sensory and perceptual dimensions associated with the five senses (16). Overall, the word classification rate of LIWC was 90.84%.

### Statistical analysis

A general linear model (GLM) repeated measure analysis was used to detect within-subject differences in the psychological process categories of the texts. The GLM analysis can be used to evaluate the variance of between-subject and within-subject factors, and it was thus used in the present study to compare the frequencies of six psychological process categories written by a group of 60 members. After an overall *F* test was executed, Fisher's least significant difference (LSD) was applied to *post hoc* multiple comparisons to measure the mean differences among categories.

To detect gender differences in the language styles, the researchers conducted a multivariate analysis of variance. Six psychological process categories and 26 subcategories constituted the dependent variables of the comparison. This multivariate procedure was used to prevent Type-I errors similar to those in multiple *t* tests.

## Results

### Differences within the psychological process categories

The GLM repeated measure analysis results revealed that cognitive words were the most prevalent, averaging 22.16%, followed by relative (13.50%), social (12.75%), biological (6.51%), affective (5.79%), and perceptual (2.86%) words (*F* (1, 5)=453.62, *P<*0.001). Fisher's LSD *post hoc* multiple comparisons (Table 1) indicated that the cognitive category substantially and statistically outperformed all five categories and that the perceptual category was least represented. These results suggested that the participants habitually exhibited cognitive processing in their observations and reflections of patients. They were mostly inclined to capture medical phenomena through logical thinking such as making cause-effect inference, decision making, and generalizations.

**Table 1:** A posteriori comparison of six psychological process categories

Word categories	M	SD	Differences	*P*
Cognitive	22.16	2.81	C>R	0.000
			C>S	0.000
			C>B	0.000
			C>A	0.000
			C>P	0.000
Relative	13.50	2.01	R>B	0.000
			R>A	0.000
			R>P	0.000
			R<C	0.000
Social	12.75	2.65	S>B	0.000
			S>A	0.000
			S>P	0.000
			S<C	0.000
Biological	6.51	1.64	B>P	0.000
			B<C	0.000
			B<R	0.000
			B<S	0.000
Affect	5.79	2.59	A>P	0.000
			A<C	0.000
			A<R	0.000
			A<S	0.000
Perceptual	2.86	2.98	P<C	0.000
			P<R	0.000
			P<S	0.000
			P<B	0.000
			P<A	0.000

Both the second- (relative) and third-ranked (social) words were common in the texts; however, they appeared approximately half as often as cognitive words did. The students appeared to have effectively recorded aspects of patients or diseases by using relative words that describe time, location, and the course of changes. The students also demonstrated an awareness of the patients and their families by acknowledging social interactions such as encouragement or persuasion.

Compared with the other categories, affective terms rarely occurred in the texts, suggesting that the students were not accustomed to observe the emotions of patients or reflect on the affective aspects of treatments. In contrast to our expectations, perceptual words were the least prevalent language mode used in the narrative accounts. Similarly, words denoting senses of perception such as ‘heard’ and ‘saw’ or describing visual or audio information such as ‘pale’ and ‘loud’ were not common. Without overstating the results, students may have tended to ‘think’ about what happened instead of detailing what actually happened about patients.

Among the perceptual words, the analysis indicated that the subcategory ‘feel’ registered the lowest average occurrence frequency percentage (0.49), whereas the occurrences of ‘see’ (0.91) and ‘hear’ (0.96) were comparable (*F* (1, 2)=18.63, *P<*0.001). One concern was that the medical students might detach themselves to some degree from the patients’ feelings and their own corresponding reactions.

### Gender differences in the language styles of the reflective writings

No overall multivariate effect was observed for gender regarding the composite psychological process categories and subcategories (*F*(1, 32) =0.583, *P*=0.93). However, Scheffe comparisons revealed that the female students used more words associated with positive emotions (2.90) and words related to sadness (0.50) than did the male students (2.33, 0.31, respectively). The result regarding expressions related to sadness is consistent with that of Newman et al. (19). However, male students outscored the female students only in the category of space (6.45 and 5.75, respectively), indicating that the male students exhibited a preference for recognizing and emphasizing spatial conditions, such as places and settings, in their reflective writing (Table 2). Although these gender differences in language use were statistically significant, the variations were relatively small. Cautions should be exercised when interpreting and applying these results.

**Table 2:** Means, standard deviations, and gender differences in language Use

	Female (*N*=26)		Male (*N*=34)			
				
Word categories	M	SD	M	SD	*F*	*P*
Positive emotion	2.90	0.92	2.33	1.04	4.75	0.035
Sad	0.50	0.41	0.31	0.29	4.36	0.041
Space	5.75	1.29	6.45	1.22	4.63	0.036

## Discussion

The finding that words in the cognitive category were most frequently recorded in the participants’ reflections is logical because this category fits the reflective cycle model of Gibbs (12), involving cognition-oriented components of evaluation, analysis, conclusion, and action plan in addition to description of experience and emotions. Reflection can be considered a form of mental process similar to critical thinking (20, 21). The preferences of medical students regarding the use of cognitive words were appropriate under the clinical rotation circumstances. However, compared with the other categories, affective words were disproportionately rare in the reflections. Although the students’ descriptions of both patients’ and their own affective experiences were highly emphasized, they infrequently appeared in the narrative accounts. This indicates a necessity to balance scientific-logic-based medicine and narrative-based medicine. According to Boud et al. (22), ‘reflection’ is a generic term for intellectual activities used to explore experiences; therefore, students’ deficiency in affective dimensions and emotional self-expression must be addressed in medical education. Increasing emotional self-expression, particularly in structured settings such as reflective groups and classroom activities, for health professionals is imperative. This is because emotional self-expression enhances their psychological well-being and their abilities to empathize with patients and themselves by enabling them to access and accept their feelings.

A strong tendency to use certain types of language indicated the reflectors’ preferred mode of processing their experience and constructing their narratives. Therefore, the smaller proportion of affective words may have been engendered by a combination of factors including the students’ lack of awareness of the affective domain for patients and their limited capabilities to decode and represent their emotions. In certain cases, students may ignore or bypass affective dimensions for the purpose of easing their anxieties because encountering the emotions associated with such dimensions may create discomfort or pain.

Examining what factors may influence the language style used by students in their reflective writing is highly appealing. For instance, students may replicate the characteristics and styles they have used in other writing assignments such as term papers or medical charts. How students perceive the course objectives and the reflection instructions in addition to how they set their own criteria to meet the instructor's expectations may have also influenced the outcome. Future qualitative studies focusing on these factors should provide meaningful findings.

Researchers have shown interest in the study of pedagogic issues (i.e., how empathy can be trained through reflective writing); however, studies that have focused on the relations between a person's linguistic patterns and his or her empathy are scant. We assumed that linguistic patterns might affect empathy outcomes. For example, first-person point-of-view writing is considered a means of moving emotionally closer to the ‘other’ (23). Another example is that Lord et al. (24) conducted a quantitative study and showed that language style synchrony was predictive of empathy ratings. Although we tended to assume that linguistic patterns might affect empathy outcomes, we also noticed that the relations between linguistic patterns and empathy might be bidirectional. For example, empathy might be defined as a mental capacity (i.e., perspective taking), and in such an instance, empathy may contribute to the manner in which a person expresses affects (i.e., words associated with positive/negative emotions).

The results reveal that male and female students exhibited comparable word usages in most of the linguistic categories, and this might be largely attributable to the traditional standard cognitive-scientific training and practice in medical fields. Regardless of gender, the participants shared a high degree of similarity in their psychological processes and expression of reflections, which might form the foundation for medical teamwork.

Despite the resemblance in linguistic patterns, the male students preferred describing spatial conditions. This is advantageous for capturing the circumstantial physical context related to patients and treatment. Compared with the male students, the female students recorded more of the positive emotions of the patients, their families, and themselves, and this finding is consistent with the results of Mehl and Pennebaker (25), who suggested that women refer to positive emotions more often than men do. Because words describing positive emotions are linearly related to positive health outcomes (11), male students should be encouraged to increase their awareness of positive emotions in addition to more negative ones.

We suggest that future studies demonstrate how the use of various linguistic categories is related to effective communication skills, enhanced self-awareness, and recognition of the affective domain of medical practice (26). Whether changes in language use, such as more balanced well-differentiated categories, would result in deepened critical thinking and whether empathetic understanding warrants further evaluation. In addition, the LIWC approach, which provides a quantitative method for analyzing reflective writings, warrants further examination of the overall effectiveness of reflective writing regarding the reflective capacity and narrative competency of students (26, 27). Researchers in future studies should select outcome measures such as empathy to determine how changes in linguistic patterns affect such outcomes. Furthermore, a mixed research design, combining qualitative and quantitative methods, might provide further insights into the current results.

Finally, the students’ reflective writing experience and the instructions or feedback provided for their writing works before this study were not comprehensively tracked. Therefore, our findings regarding the prevalence of the use of words belonging to various psychological process categories in addition to the effects of gender differences on word usage may reflect an accumulated outcome of the students’ total learning experience. It may not be considered the pure effect of this required course or students’ natural language styles. Moreover, whether students discussed their writing works by themselves before submitting their assignments was uncertain. The potential peer influence should be controlled or analyzed in future studies. The exclusion rate (i.e., 16%) of the total participants necessitates exploring the circumstance or factors attributed to students’ inability to follow writing instructions in the future. A controlled study that entails comparing the pre- and postwriting styles as well as teaching approaches that involve excluding less invalid reflective texts is also required.

## Conclusions

While maintaining their cognitive comprehension of health-care phenomena, medical students should be encouraged to enhance their empathetic understanding of the psychosocial issues in patients and illness by becoming aware of and expressing their affective experiences. Moreover, perceptions provide the basis for further reflection; therefore, enhancing the quality and quantity of perceptions is a critical step in forming solid reflections. For training purposes, we suggest that medical teachers use structured activities that enable students to see and hear what happens in the interactions of patients and their family members. Finally, clinical teachers can help students discern their strengths and weaknesses in various psychological process modes. Male and female students can mutually benefit from one another's advantages and determine whether to highlight certain aspects of their reflections by choosing to use various types of language. Studies on the psychology of word usage in narrative medicine remain rare, particularly in Taiwan and Asia, and additional studies should be undertaken in the near future.
